# Knowledge, Awareness, and Perceptions Towards Antibiotic Use, Resistance, and Antimicrobial Stewardship Among Final-Year Medical and Pharmacy Students in Saudi Arabia

**DOI:** 10.3390/antibiotics14020116

**Published:** 2025-01-22

**Authors:** Abdullah A. Alshehri, Wael Y. Khawagi

**Affiliations:** Department of Clinical Pharmacy, College of Pharmacy, Taif University, Taif 21944, Saudi Arabia; w.khawagi@tu.edu.sa

**Keywords:** antibiotic resistance, antimicrobial stewardship, antibiotic use, prescribing practices, healthcare education, knowledge assessment, awareness, perceptions, Saudi Arabia

## Abstract

**Background/Objectives**: Antibiotic resistance (ABR) is a global crisis leading to increased mortality and economic burden. Antimicrobial stewardship (AMS) promotes responsible antibiotic use and prescribing practices to combat ABR. This study assessed the knowledge, awareness, and perceptions of final-year medical and pharmacy students in Saudi Arabia regarding antibiotic use, ABR, and AMS. **Methods**: A national cross-sectional survey was conducted from January to April 2024 using a 49-item questionnaire. The survey assessed knowledge of antibiotic use, ABR, and AMS using predefined scoring, while perceptions were evaluated with a 5-point Likert scale. Data were analyzed using descriptive statistics and Chi-square tests to explore associations between independent variables and outcomes. A logistic regression analysis identified factors associated with good knowledge, and results were reported as adjusted odds ratios (aOR) with 95% confidence intervals (CI). **Results:** Among 197 participants from 26 Saudi universities, pharmacy students comprised 73.1% of the sample. Good knowledge was observed in 44.7% for antibiotic use, 47.7% for ABR, and 52.8% for AMS. However, 28.4% lacked awareness of AMS, and 5.6% were unaware of ABR. Pharmacy students demonstrated significantly higher knowledge and confidence in AMS and antibiotic handling compared to medical students (*p* < 0.001). Higher knowledge was also associated with the university’s region and prior education on resistance or AMS during college. **Conclusions**: This study highlights significant gaps in knowledge and awareness of antibiotic use, ABR, and AMS among final-year medical and pharmacy students in Saudi Arabia. The findings emphasize the need for targeted educational strategies, standardized curricula, and enhanced AMS training to better prepare future healthcare professionals to combat ABR effectively.

## 1. Introduction

Antibiotic resistance (ABR) is a critical global health threat, primarily driven by the misuse and overuse of antibiotics, leading to multidrug-resistant pathogens [1,2]. In 2021, ABR contributed to over 4.7 million deaths worldwide, including 1.2 million directly attributable to resistant pathogens [3]. Without intervention, drug-resistant infections could cause over 39 million deaths by 2050, with annual deaths rising by 70% to 1.91 million [3]. The economic burden is equally alarming, with ABR adding over $2 billion in annual global costs, projected to reach $1 trillion by 2050 [4,5,6]. In Saudi Arabia, the prevalence of multidrug-resistant pathogens, including Gram-negative bacteria and methicillin-resistant Staphylococcus aureus (MRSA), in healthcare-associated infections underscores the urgency for effective global and local interventions to combat ABR [7,8].

Antimicrobial stewardship (AMS) has proven to be a key strategy in combating ABR by promoting the responsible use of antimicrobials, optimizing patient outcomes, and reducing resistance rates and healthcare costs [9,10]. Successful global AMS initiatives include the UK’s ‘Start Smart–Then Focus’, the U.S. CDC’s Core Elements of Stewardship, Sweden’s ‘Strama’, and Australia’s National Antimicrobial resistance (AMR) Strategy, all of which emphasize responsible antibiotic use, national coordination, and public awareness [11,12,13,14]. AMS initiatives include public awareness, professional education, and coordinated programs aimed at reducing inappropriate antibiotic use and improving clinical practices [15,16]. The World Health Organization (WHO) emphasizes enhancing education and awareness among healthcare professionals as a cornerstone of its global action plan to mitigate ABR [17,18].

Central to the success of AMS are healthcare providers, particularly physicians and pharmacists, who play pivotal roles in its implementation [19,20]. They are responsible for optimizing antimicrobial prescribing, educating patients, and ensuring adherence to evidence-based guidelines. Despite their pivotal role, irrational antibiotic use persists, with studies indicating that 20% to 50% of hospital prescriptions and over half of community-dispensed antibiotics can be considered inappropriate [21,22]. In developing countries, these challenges are coupled with inadequate knowledge among healthcare professionals, insufficient training, limited diagnostic facilities, and the absence of standardized treatment guidelines [23,24,25].

Equally important in the fight against ABR are final-year medical and pharmacy students, who represent a crucial cohort transitioning from academic learning to clinical practice [26,27]. This phase is instrumental in shaping their understanding of ABR, AMS principles, and appropriate antibiotic use. Research highlights the significant influence of undergraduate education on future healthcare professionals’ attitudes and practices concerning antibiotic use [28]. However, knowledge gaps in infection management and the implementation of AMS persist among graduating students [29,30]. While several studies have examined the knowledge and awareness of final-year students in various countries [31,32,33,34], data from Saudi Arabia remain limited. Existing studies have largely focused on AMS knowledge and perceptions at individual institutions [23,35]. These localized findings highlight the necessity for broader, nationwide research to comprehensively assess students’ readiness to address ABR.

Saudi Arabia has prioritized combating ABR through its National Action Plan (NAP), which aligns with the World Health Organization’s Global Action Plan and emphasizes the need for enhanced education and awareness regarding ABR and AMS across healthcare professions [17,36]. This national commitment underscores the importance of evaluating the preparedness of future healthcare professionals to contribute effectively to these efforts. Therefore, this study aims to evaluate the knowledge, awareness, and perceptions of final-year medical and pharmacy students across Saudi Arabia regarding antibiotic use, ABR, and AMS. The findings will identify strengths, areas for improvement, and prevailing perceptions, and will inform targeted educational interventions to better prepare future healthcare professionals to combat ABR and safeguard the effectiveness of antimicrobials for future generations.

## 2. Results

### 2.1. Demographic and Educational Characteristics

A total of 197 participants were included in this study. The mean age of the participants was 23.6 ± 1.3 years, with the majority being male (59.4%). Regarding specialty, most participants were enrolled in pharmacy programs (73.1%), while the remainder were in medical programs (26.9%). When examining academic performance according to students’ grade point average (GPA), nearly half of the participants (47.7%) were categorized as Excellent, followed by 34% as Very Good, 16.3% as Good, and 2% as Accepted. Approximately half of the participants (50.3%) reported attending training or specialized education in infectious diseases beyond their college program. The participants were distributed across 26 universities in Saudi Arabia. When categorized by university region, the majority of the participants were from the Western region (55.8%), followed by the Central region (21.3%), Southern region (9.7%), Northern region (8.1%), and the Eastern region (5.1%). The demographic and educational characteristics of the participants are summarized in Table 1.

### 2.2. Knowledge and Awareness Across Antibiotic Use and Prescribing Practices, Antibiotic Resistance, and Antimicrobial Stewardship

**Figure 1:**
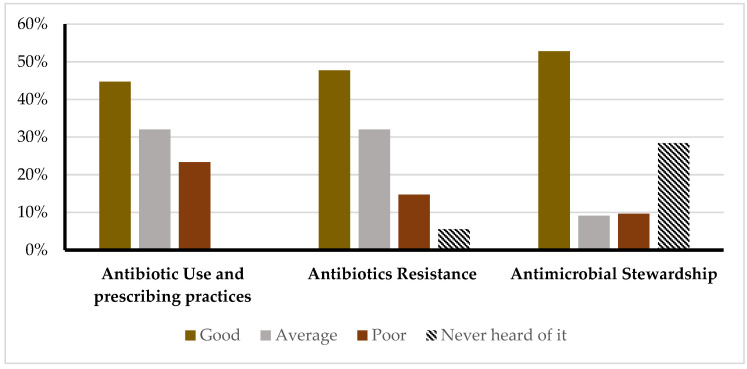
Knowledge and awareness across antibiotic use and prescribing practices, antibiotic resistance, and antimicrobial stewardship.

The participants were assessed for their knowledge levels across antibiotic use and prescribing practices, antibiotic resistance, and AMS programs. The distribution of knowledge and awareness across the three domains is illustrated in Figure 1.

For antibiotic use, 44.7% demonstrated a strong understanding, 32% moderate, and 23.3% limited knowledge. Regarding antibiotic resistance, 47.7% had a good grasp, 32% showed a fair understanding, 14.7% had weak knowledge, and 5.6% had never heard of the concept. For antimicrobial stewardship programs, 52.8% had high knowledge, 9.1% had moderate knowledge, and 9.7% had poor knowledge, while 28.4% were unfamiliar with the concept prior to the study.

### 2.3. Factors Associated with Knowledge of Antibiotic Use and Prescribing Practices, Antibiotic Resistance, and Antimicrobial Stewardship

The study identified several factors associated with the participants’ knowledge across all domains. The Chi-square analysis revealed that university region was significantly associated with knowledge of antibiotic use (*p* = 0.018) and AMS programs (*p* = 0.023), while students’ specialty was significantly associated with knowledge in all areas (*p* < 0.001). Knowledge of antibiotic use and resistance was also linked to being taught about resistance in college (*p* = 0.003 and *p* = 0.002, respectively), and knowledge of AMS programs was associated with prior education on AMS (*p* < 0.001). However, attending formal training or specialized education in infectious diseases beyond the college program did not show a significant association with knowledge scores in any domain (Table 2).

The multivariate logistic regression further explored these associations. For antibiotic use and prescribing practices, the participants from the Eastern region were more likely to demonstrate good knowledge compared to those from the Central region (aOR = 5.21, 95% CI: 1.05–25.93, *p* = 0.044). Similarly, pharmacy students were significantly more likely to have good knowledge compared to medical students across all domains, with the strongest association observed for AMS programs (aOR = 7.66, 95% CI: 3.26–18.00, and *p* < 0.001). Being taught about antibiotic resistance in college was also a significant predictor of good knowledge for all domains, particularly for antibiotic use (aOR = 3.48, 95% CI: 1.43–8.47, and *p* = 0.006) and AMS programs (aOR = 3.32, 95% CI: 1.23–8.92, and *p* = 0.017). Additionally, being taught about AMS in college was strongly associated with good knowledge of AMS programs (aOR = 4.20, 95% CI: 1.85–9.55, and *p* = 0.001) (Table 3).

### 2.4. Students’ Perceptions

#### 2.4.1. Confidence in AMS and Antibiotic Resistance

**Figure 2:**
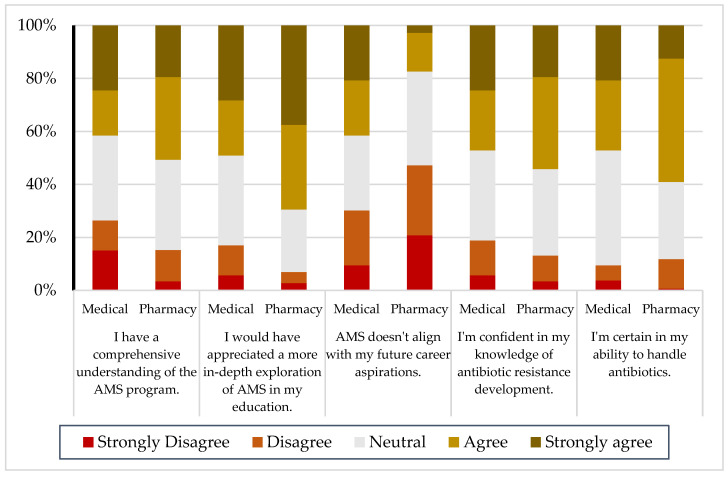
Distribution of medical and pharmacy students’ responses regarding their perceptions of AMS, ABR, and antibiotic handling.

The survey revealed differences between the medical and pharmacy students in their confidence regarding AMS, antibiotic resistance, and handling antibiotics. While both groups demonstrated notable levels of confidence, the pharmacy students generally reported a higher confidence in their knowledge and abilities. For instance, 50.7% of the pharmacy students and 41.5% of the medical students agreed or strongly agreed that they had a comprehensive understanding of AMS. Similarly, the confidence in understanding antibiotic resistance was slightly higher among the pharmacy students (54.2%) compared to the medical students (47.2%). The confidence in handling antibiotics followed a similar pattern, with 59% of the pharmacy students expressing confidence compared to 47.2% of the medical students (Figure 2).

#### 2.4.2. Perceived Need for AMS Education

Both groups strongly recognized the need for enhanced AMS education, emphasizing the importance of incorporating more comprehensive and practical AMS training into curricula. Among the pharmacy students, 69.5% strongly agreed or agreed that more in-depth AMS education was needed, compared to 49.1% of the medical students (Figure 2).

#### 2.4.3. Career Alignment

Both groups demonstrated varying degrees of alignment between AMS and their career aspirations. Pharmacy students were less likely to agree with the statement “AMS doesn’t align with my future career aspirations”, with 17.4% agreeing or strongly agreeing compared to 41.5% of the medical students. Conversely, 47.2% of the pharmacy students and 30.2% of the medical students disagreed or strongly disagreed with the statement, suggesting a stronger alignment of AMS with the career goals of pharmacy students. Neutral responses were frequent across all the statements among both groups, particularly among the medical students, with up to 43.4% indicating neutrality on various items, highlighting a level of uncertainty in their self-assessments (Figure 2).

## 3. Discussion

This national study assessed the knowledge, awareness, and perceptions of final-year medical and pharmacy students in Saudi Arabia regarding antibiotic use, resistance, and AMS programs. Knowledge levels varied, with approximately half of the students demonstrating a good understanding of these topics. However, a significant proportion lacked awareness of AMS programs and antibiotic resistance. Statistically significant associations were identified between good knowledge and factors such as the region, healthcare field, and being taught about resistance and stewardship in college. The pharmacy students reported a greater confidence in handling antibiotics, understanding resistance, and engaging with AMS, while the medical students presented more uncertainty. Both groups emphasized the need for enhanced AMS education, highlighting gaps in current education and emphasizing the importance of targeted strategies to address antibiotic resistance.

Our findings on knowledge align with existing literature but reveal critical gaps. Regarding antibiotic use and prescribing practices, a significant proportion of the participants demonstrated a strong understanding, consistent with studies highlighting good baseline knowledge among pharmacy students and healthcare providers, though gaps in application remain [37,38]. For antibiotic resistance, while many participants displayed good knowledge, a small portion was unaware of the concept. Similarly, nursing and healthcare students in Rwanda and Spain have shown low levels of AMR knowledge despite recognizing its importance [39,40]. The differences may stem from variations in healthcare systems, such as access to AMS resources, national AMR strategies, and emphasis on undergraduate education. The awareness of AMS in our study was variable, with many participants showing high knowledge but a notable number lacking any prior awareness. These findings mirror gaps identified among healthcare workers in Malawi and Italy, emphasizing the global need for improved AMS education [41,42].

Regional disparities were evident, with Eastern-region participants demonstrating better knowledge. Geographic differences may reflect variations in resources, faculty expertise, the availability of specialized training, and cultural factors such as attitudes toward education [43]. Institutions in regions with advanced healthcare systems or partnerships with teaching hospitals may offer better practical training. These findings suggest the need for national oversight to ensure a consistent educational quality across universities [44,45]. Collaborative initiatives, such as national workshops and training programs, could address these disparities.

Formal education on ABR and AMS during college was a strong predictor of knowledge, consistent with previous studies emphasizing the critical role of undergraduate curricula in building foundational understanding [46,47]. In contrast, specialized training beyond college did not show a significant impact on knowledge levels. These events typically offer general overviews rather than in-depth learning or practical application, reducing their ability to enhance foundational knowledge [48,49,50,51]. This highlights the importance of embedding AMS content early in education, as well-designed college curricula with clear objectives and structured content effectively equip students with a solid foundation in AMS principles [52,53,54,55,56]. In addition, to maximize the benefits of specialized training beyond college, future initiatives should focus on structured, standardized training programs that include clear learning objectives, practical components, and robust assessment methods. These efforts would complement existing educational curricula and enhance students’ understanding of antimicrobial resistance (ABR) and AMS [57].

The pharmacy students outperformed the medical students across all the domains, particularly in AMS knowledge, confidence, and career alignment, aligning with evidence that pharmacy curricula often incorporate more AMS-focused content [31,58,59]. Courses in pharmacotherapy and hospital pharmacy place a strong emphasis on the pharmacist’s role in antimicrobial stewardship, encompassing responsibilities such as monitoring antimicrobial use, providing education to healthcare teams, and ensuring adherence to evidence-based guidelines. These disparities highlight the need for a more standardized and comprehensive approach [60,61]. Curricula should include more practical, case-based learning opportunities, such as problem-based scenarios and simulated patient interactions, to bridge gaps in understanding and application [53,62]. In addition, efforts should focus on strengthening medical students’ training in antibiotic resistance and AMS by incorporating dedicated AMS modules, integrating these topics into clinical rotations, and involving students in real-time antimicrobial decision-making alongside healthcare teams. Structured workshops, interdisciplinary training sessions, and the use of e-learning platforms can further reinforce these topics, ensuring their relevance to clinical practice.

Future research should focus on the long-term impact of interventions aimed at improving AMS education, particularly in assessing their effects on knowledge retention, attitudes, and clinical practices over time. Additionally, exploring the integration of AMS topics into medical and pharmacy curricula in Saudi Arabia is crucial. Studies should investigate which specific courses include AMS content and whether AMS is obligatorily taught in these programs. This would help identify gaps in curriculum design and opportunities for standardization across institutions. More in-depth qualitative studies involving students, educators, and academic institutions are necessary to understand the challenges and facilitators of effective AMS education. These studies could provide valuable insights into how curricula are implemented and received, and how they can be optimized to meet the needs of diverse learner groups. Expanding research to include other healthcare disciplines, such as nursing and allied health professionals, would provide a more holistic understanding of AMS education across interprofessional teams. Furthermore, it is important to explore innovative delivery methods for AMS education, such as simulation-based training, e-learning platforms, and gamified approaches. Research should investigate the effectiveness of these methods compared to traditional teaching strategies in enhancing engagement, knowledge acquisition, and practical skills [63,64].

This study has several strengths and limitations. This is the first study in Saudi Arabia to comprehensively assess the knowledge, awareness, and perceptions of final-year medical and pharmacy students regarding antibiotic use, ABR and AMS programs. The sample size in this study was larger than those in similar studies [65,66], enhancing the reliability and robustness of the findings. However, several limitations must be considered. The use of snowball sampling made it impossible to calculate a precise response rate, which is a limitation inherent to this method. Additionally, snowball sampling may introduce selection bias, as students with greater interest or knowledge in antibiotic resistance may be more likely to participate, potentially overestimating awareness levels. The sample was also skewed, with about half of the participants from universities in the western region, which may limit the generalizability of the findings to other areas in Saudi Arabia. The nature of the questionnaire itself could also contribute to recall and response bias, as participants might overestimate their knowledge or provide socially desirable answers. These factors could affect the overall applicability and accuracy of the findings.

## 4. Materials and Methods

### 4.1. Study Design and Participants

A national cross-sectional survey was conducted using an online, self-administered questionnaire distributed over a five-month period (January–April 2024). Final-year undergraduate medical and pharmacy students enrolled in universities across Saudi Arabia were eligible to participate. Students not in their final year or enrolled in non-medical or non-pharmacy programs were excluded from the study. This design was selected as it is well suited for capturing a snapshot of knowledge, attitudes, and practices at a single point in time, aligning with the study’s objective [67,68]. 

### 4.2. Questionnaire Development

The questionnaire was developed based on previously validated studies to assess students’ knowledge and perceptions [32,65,66,69]. The questionnaire was refined with input from academic pharmacists holding PhDs in pharmacy practice to ensure comprehensive coverage, cultural relevance, and alignment with the Saudi educational system, with experts familiar with local educational curricula and healthcare practices reviewing the questions for appropriateness. The questionnaire was created using Google Forms in English and included a preface outlining the study objectives, confidentiality details, researcher contact information, and a consent checkbox.

Comprising 49 closed-ended questions, the questionnaire had five parts. Part 1 collected demographic details such as gender, age, university, and involvement in training or education related to infectious diseases. Part 2 assessed knowledge of antibiotic use and prescribing practices with 13 questions, while Part 3 evaluated knowledge of antibiotic resistance through 10 questions, starting with whether participants had heard of it. Part 4 focused on AMS knowledge with 10 questions, beginning with awareness of AMS programs. Part 5 explored perceptions of AMS, confidence in knowledge of antibiotic resistance, and its career relevance.

The responses in Parts 2–4 were scored as “Yes”, “No”, or “Do not know”, with one point for correct answers and zero for incorrect or “Do not know” responses. For Part 2 (antibiotic use and prescribing practices), scores of 0 to 5 were classified as poor, 6 to 9 as average, and 10 to 13 as good. For Part 3 (antibiotic resistance), scores of 0 to 4 were classified as poor, 5 to 7 as average, and 8 to 10 as good. For Part 4 (AMS program), scores of 0 to 4 were considered poor, 5 to 7 as average, and 8 to 10 as good. Part 5 used a 5-point Likert scale from “Strongly agree” to “Strongly disagree”. The survey took 7–10 min to complete (Supplementary File S1).

### 4.3. Piloting the Questionnaire

To ensure clarity and effectiveness, a pilot study was conducted with 12 targeted students to evaluate its readability, comprehensibility, and feasibility. Based on their feedback, minor adjustments were made to improve the wording of certain items. The participants in the pilot study were excluded from the final analysis.

### 4.4. Sample Recruitment and Data Collection

The online questionnaire was disseminated to final-year undergraduate medical and pharmacy students in Saudi Arabia through social media platforms (WhatsApp) and professional networks of student associations. Snowball sampling was employed to maximize reach, where the participants were encouraged to share the questionnaire link with their peers and colleagues. It is important to note that snowball sampling may introduce selection bias, as students with greater interest or knowledge in antibiotic resistance may be more likely to participate.

### 4.5. Statistical Analysis

Data were analyzed using Stata (version 16). Descriptive statistics summarized the participant characteristics and outcome measures. The survey was designed with forced questions, ensuring that all required fields were completed, thereby eliminating the possibility of missing data during the analysis. Logistic regression was employed to evaluate the associations between independent variables and the likelihood of the participants being classified as having “good” knowledge in each binary outcome (antibiotic use and prescribing practices, antibiotic resistance, and AMS program). The independent variables included demographic characteristics and factors related to education and training (e.g., whether resistance and AMS were taught in college).

A forward stepwise logistic regression approach was used. The variables were first evaluated using Chi-square tests for their association with each outcome. Only variables with a *p*-value < 0.05 in the Chi-square tests were included in the model. All the statistical tests were two-sided, and a *p*-value < 0.05 was considered statistically significant. The results are presented as adjusted odds ratios (aOR) with 95% confidence intervals (CI).

### 4.6. Ethical Considerations

Ethical approval has been obtained from the ethical committee at Taif University (Application No. 45-075, Date: 26 November 2023). To ensure privacy and confidentiality, no personal information was collected from the questionnaire participants. Participation in the study was voluntary, and the participants had the right to withdraw at any point without any consequence. Data access was strictly limited to the research team, adhering to ethical standards, and maintaining rigorous control over data handling throughout the study.

## 5. Conclusions

This study highlights critical gaps in the knowledge, awareness, and perceptions of final-year medical and pharmacy students in Saudi Arabia regarding antibiotic use, resistance, and AMS programs. While nearly half demonstrated good knowledge, the pharmacy students consistently outperformed the medical students, emphasizing the need for standardized and comprehensive AMS education across disciplines. Key predictors of knowledge included university region and formal education on AMS and resistance, underscoring the importance of embedding structured AMS content into undergraduate curricula. To address these gaps, integrating AMS education into all healthcare programs, along with targeted strategies, practical learning, and interdisciplinary workshops, will improve knowledge retention and better prepare healthcare professionals to combat antibiotic resistance.

## Figures and Tables

**Table 1:** Demographic and Educational Characteristics of Participants.

Characteristic		N (%)/Mean ± SD
Gender		
	Female	80 (40.6)
	Male	117 (59.4)
Age *		23.6 ± 1.3
Healthcare Field		
	Medical	53 (26.9)
	Pharmacy	144 (73.1)
Student Level		
	Excellent	94 (47.7)
	Very Good	67 (34)
	Good	32 (16.3)
	Accepted	4 (2)
Attend Training or Specialized Education in Infectious Diseases		
	No	98 (49.7)
	Yes	99 (50.3)
Taught about antibiotic resistance in college		
	No	24 (12.2)
	Yes	158 (80.2)
	I do not know	15 (7.6)
Taught about AMS program in college		
	No	37 (18.8)
	Yes	132 (67)
	I do not know	28 (14.2)
University Region		
	Central	42 (21.3)
	Eastern	10 (5.1)
	Northern	16 (8.1)
	Southern	19 (9.7)
	Western	110 (55.8)

* Mean ± SD for continuous variables.

**Table 2:** Chi-Square Correlation Results for Factors Associated with Knowledge of Antibiotic Use and Prescribing Practices, Antibiotic Resistance, and Antimicrobial Stewardship.

	Antibiotic Use and Prescribing Practices	Antibiotic Resistance	Antimicrobial Stewardship
Gender	0.213	0.128	0.094
Region	0.018 *	0.463	0.023 *
Healthcare field	<0.001 *	<0.001 *	<0.001 *
GPA category	0.872	0.952	0.161
Attend training or specialized education in infectious diseases	0.824	0.555	0.286
Taught about antibiotic resistance in college	0.003	0.002	<0.001 *
Taught about AMS in college	0.543	0.894	<0.001 *

* Indicates statistically significant associations (*p* < 0.05). AMS: antimicrobial stewardship.

**Table 3:** Adjusted Multivariate Logistic Regression Analysis of Significant Characteristics for Knowledge Scores in Antibiotic Use and Prescribing Practices, Antibiotic Resistance, and Antimicrobial Stewardship Among Final-Year Medical and Pharmacy Students.

Characteristic		Antibiotic Use and Prescribing Practices			Antibiotic Resistance			Antimicrobial Stewardship		
		% *	aOR (95% CI)	*p*-Value	% *	aOR (95% CI)	*p*-Value	% *	aOR (95% CI)	*p*-Value
University region										
	Central	30.9	Reference		-	-	-	35.7	Reference	
	Northern	75.0	4.32 (1.12–16.61)	0.033	-	-	-	81.2	5.25 (1.12–24.66)	0.036
	Southern	47.3	1.92 (0.59–6.23)	0.279	-	-	-	52.6	2.17 (0.61–7.70)	0.232
	Western	42.7	1.89 (0.84–4.25)	0.125	-	-	-	56.3	2.43 (1.00–5.93)	0.051
	Eastern	70.0	5.21 (1.05–25.93)	0.044	-	-	-	40.0	0.60 (0.12–2.94)	0.529
Healthcare field										
	Medicine	18.9	Reference		19.61	Reference		18.9	Reference	
	Pharmacy	54.2	4.53 (2.03–10.12)	<0.001	62.22	6.44 (2.93–14.14)	<0.001	65.3	7.66 (3.26–18.00)	<0.001
Taught about resistance in college										
	No	20.5	Reference		25.0	Reference		23.1	Reference	
	Yes	50.6	3.48 (1.43–8.47)	0.006	55.8	3.44 (1.38–8.57)	0.008	60.1	3.32 (1.23–8.92)	0.017
Taught about AMS in college										
	No	-	-	-	-	-	-	26.1	Reference	
	Yes	-	-	-	-	-	-	65.9	4.20 (1.85–9.55)	0.001

* The percentages represent the proportion of participants categorized as “good” for each knowledge score. aOR: adjusted odds ratio; CI: confidence interval; and AMS: antimicrobial stewardship.

## Data Availability

The datasets used and analyzed during the current study are available from the corresponding author on reasonable request.

## Supplementary Materials

The following supporting information can be downloaded at https://www.mdpi.com/article/10.3390/antibiotics14020116/s1: Supplementary File S1: Questionnaire.
