# Supplementary material for: Knowledge, Awareness, and Perceptions Towards Antibiotic Use, Resistance, and Antimicrobial Stewardship Among Final-Year Medical and Pharmacy Students in Saudi Arabia
Source: Antibiotics (Basel). 2025 Jan 22;14(2):116. doi: 10.3390/antibiotics14020116 (PMC11852200; doi:10.3390/antibiotics14020116)
Supplement: Supplementary file 1 [file antibiotics-14-00116-s001.zip › antibiotics-3408182-supplementary.pdf]

Knowledge, Awareness, and Perceptions towards Antibiotic Use, Resistance, and Antimicrobial Stewardship Among Final-Year Medical and Pharmacy Students in Saudi Arabia

We are a research group at College of Pharmacy, Taif University that is conducting a cross-sectional study with undergraduate Medicine and Pharmacy students in Saudi Arabia. This survey aims to Evaluate the knowledge, awareness and perceptions of future physicians and pharmacists regarding the risks of antibiotics resistance, specifically focusing on their understanding and sense of responsibility towards antimicrobial stewardship.

The survey should take about 10 minutes to complete. All information you have provided will be anonymised and kept confidential. Access to the data will be limited to the research team and available to authorised researchers only. You can withdraw from the survey anytime, without giving any explanation.

If you have any further questions, please do not hesitate to contact the principal investigators via the email address provided. Dr. Abdullah A Alshehri (a.aalshehri@tu.edu.sa) OR Dr. Wael Y Khawagi (w.khawagi@tu.edu.sa)

Please read the information above carefully and if you are happy to participate put your initials in the right-hand box.

I confirm I have read the information above and consent to participate. ☐

Part1. Demographics

1. Gender:
   - o Male
   - o Female

2. Age: ______________

3. University affiliation: ____________________

4. Academic program (healthcare filed):
   - o Medical
   - o Pharm.D.
   - o B.Pharm.

5. Year:
   - o 5th
   - o 6th
   - o 7th

6. Student’s level (GPA):
   - Excellent [Out of 4: 3.5-4 , Out of 5:4.5-5]
   - Very Good [Out of 4: 2.75-3.49 , Out of 5: 4-4.49]
   - Good [Out of 4: 1.75-2.74 , Out of 5: 3.25-3.99]
   - Accepted [Out of 4: 1.1-1.74 , Out of 5: 2.5-3.24]

7. Have you received/attend any formal training or specialized education in Infectious Diseases beyond your college program?
   - Yes
   - No

8. Have you ever been taught antibiotic resistance in your college program?
   - Yes
   - No

9. Was antibiotic stewardship taught in your college program?
   - Yes
   - No

Part2. Understanding of Antibiotic Use and Prescribing Practises

Please tick in the correct column from options ‘yes’, ‘no’, and ‘do not know’.

No.	Questions	Yes	No	Don’t know
1	Antibiotics are the most commonly prescribed anti-infective agents.			
2	Treating common cold with antibiotics speed up recovery			
3	Antibiotics should be prescribed as preventive measures to fight against future microbial attacks			
4	Influenza can be treated with antibiotics			
5	Antibiotics might develop allergy in susceptible individuals.			
6	Antibiotics can be obtained without a prescription in Saudi Arabia			
7	Antibiotics are the first line of treatment for common sore throat			
8	Cefotaxime belongs to the third-generation cephalosporins			
9	The only penicillin agent has antipseudomonal activity is ampicillin			
10	Penicillin G benzathine is the drug of choice for syphilis			
11	Salbutamol is an antibiotic used in treating upper respiratory infections			
12	The invasive phase of active TB regimen include 4 drugs for 4 months			

13	Cefoxitin is the only beta lactam antibiotics with proven activity against MRSA			
	Score			

Part3. Understanding the knowledge of AR.

Please tick in the correct column from options ‘yes’, ‘no’, and ‘do not know’.

Questions	Yes	No
Have you ever heard of antibiotic resistance?		

If Yes go the to next questions of part 3

No.	Questions	Yes	No	Don’t know
1	Antibiotic resistance can be developed due to incorrect prescription or diagnosis of antibiotics.			
2	Antibiotic resistance can be developed due to insufficient dose/frequency.			
3	Antibiotic resistance can be developed due to prolonged exposure to antibiotics.			
4	Antibiotic resistance cannot be inherited among bacteria.			
5	Bacteria which are resistant to antibiotics cannot be spread from person to person.			
6	Bacteria which are resistant to antibiotics cannot be transmitted through contaminated food or water			
7	*E. coli* is resistant to vancomycin because it cannot penetrate the cell wall.			
8	Broad-spectrum antibiotics does not cause supra-infection.			
9	Bacteria cannot transfer resistant gene among each other.			
10	Some bacteria produce enzymes that inhibit specific antibiotics.			
	Score			

Part4. Understanding of AMS program

Please tick in the correct column from options ‘yes’, ‘no’, and ‘do not know’.

Questions	Yes	No
Have you ever heard of Antimicrobial stewardship program?		

If Yes go the to next questions

No.	Questions	Yes	No	Don’t know
1	Antimicrobial stewardship (AMS) program aims to educate healthcare providers and patients about inappropriate use of antibiotics			
2	AMS program can maximize antibiotic resistance phenomenon			

3	AMS program depends on physicians only			
4	AMS covers choosing antimicrobials appropriately			
5	AMS covers selecting suitable dosing regimens for antimicrobials			
6	AMS covers selecting suitable routes of administration for antimicrobials			
7	AMS covers choosing appropriate duration of therapy for antimicrobial drugs			
8	AMS covers switching from IV to oral antibiotic			
9	AMS program enhances the cost-effectiveness of antimicrobial therapy			
10	AMS program promotes rational use of antimicrobial therapy			
Score				

Part5. students' perceptions

Please tick in the correct column from options ‘Strongly Agree, ‘Agree, Neutral’, Disagree, and Strongly Disagree

No.	Questions	Strongly Agree	Agree	Neutral	Disagree	Strongly Disagree
1	I have a comprehensive understanding of the AMS program.					
2	I would have appreciated a more in-depth exploration of AMS in my education.					
3	AMS doesn't align with my future career aspirations.					
4	I'm confident in my knowledge of antibiotic resistance development.					
5	I'm certain in my ability to handle antibiotics.					

THANK YOU FOR COMPLETING THIS QUESTIONNAIRE
